# Supplementary material for: Towards a COVID-19 symptom triad: The importance of symptom constellations in the SARS-CoV-2 pandemic
Source: PLoS One. 2021 Nov 22;16(11):e0258649. doi: 10.1371/journal.pone.0258649 (PMC8608328; doi:10.1371/journal.pone.0258649)
Supplement: S1 Fig — (DOCX) [file pone.0258649.s001.docx]

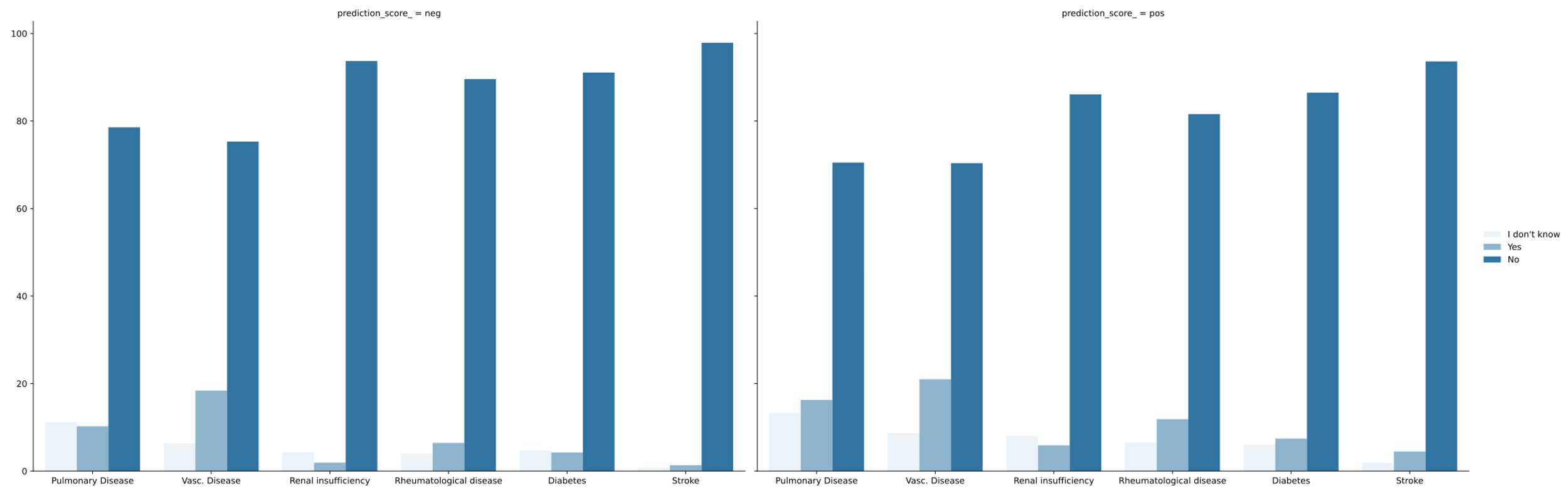


S1 Fig. Comparison of users with confirmed and without confirmed contact regarding risk factors.
