# Supplementary material for: Towards a COVID-19 symptom triad: The importance of symptom constellations in the SARS-CoV-2 pandemic
Source: PLoS One. 2021 Nov 22;16(11):e0258649. doi: 10.1371/journal.pone.0258649 (PMC8608328; doi:10.1371/journal.pone.0258649)
Supplement: S1 Table — (DOCX) [file pone.0258649.s004.docx]

**S1 Table. Survey questions of the COVID-Online application, divided by languages (German, English, French).**

| **Allgemeine Angaben** | **General information** | **Informations générales** |
| --- | --- | --- |
| 1. Geschlecht | 1. Gender | 1. Sexe |
| 1. Weiblich 2. Männlich 3. Divers | 1. Female 2. Male 3. Diverse | 1. Féminin 2. Masculin 3. Divers |
| 2. Größe | 2. Height | 2. Taille |
| 1. Kleiner als 1,50 m 2. 1,51 bis 1,60 m 3. 1,61 bis 1,70 m 4. 1,71 bis 1,80 m 5. 1,81 bis 1,90 m 6. 1,91 bis 2,00 m 7. Über 2,00 m | 1. < 1.50 m 2. 1.51-1.60 m 3. 1.61-1.70 m 4. 1.71-1.80 m 5. 1.81-1.90 m 6. 1.91-2.00 m 7. > 2.00 m | 1. < 1.50 m 2. 1.51-1.60 m 3. 1.61-1.70 m 4. 1.71-1.80 m 5. 1.81-1.90 m 6. 1.91-2.00 m 7. > 2.00 m |
| 3. Gewicht | 3. Body weight | 3. Poids |
| 1. Weniger als 60kg 2. 61-70 kg 3. 71-80 kg 4. 81-90 kg 5. 91-100 kg 6. 101-110 kg 7. 111-120kg 8. Mehr als 120kg | 1. < 60 kg 2. 61-70 kg 3. 71-80 kg 4. 81-90 kg 5. 91-100 kg 6. 101-110 kg 7. 111-120 kg 8. > 120 kg | 1. < 60 kg 2. 61-70 kg 3. 71-80 kg 4. 81-90 kg 5. 91-100 kg 6. 101-110 kg 7. 111-120 kg 8. > 120 kg |
| 4. Wie alt sind Sie? | 4. How old are you? | 4. Quel age avez-vous? |
| 1. Jünger als 20 2. 20-30 3. 31-40 4. 41-50 5. 51-60 6. 61-70 7. 71-80 8. Über 80 | 1. < 20 2. 20-30 3. 31-40 4. 41-50 5. 51-60 6. 61-70 7. 71-80 8. > 80 | 1. < 20 2. 20-30 3. 31-40 4. 41-50 5. 51-60 6. 61-70 7. 71-80 8. > 80 |
| **Krankheitsspezifische Fragen**  Scoring für Wahrscheinlichkeit einer Covid-19-Infektion | **Disease-specific questions**  Scoring for the probability of Covid-19 infection | **Questions spécifiques à la maladie**  Score pour la probabilité d'infection Covid-19 |
| 5. Hatten Sie engen Kontakt zu einem bestätigten Coronafall? | 5. Did you have close contact with a confirmed Corona case? | 5. Étiez-vous en contact étroit avec un cas Corona confirmé? |
| **a.** Ja 🡪 Wann war der letzte Kontakt? Anmerkung: Bitte nehmen Sie Kontakt zur Hotline des regionalen Gesundheitsamtes auf. Gesundheitsamt Marburg: Tel.: 06421-40544  1. innerhalb der letzten 14 Tage  2. länger als 14 Tage her  **b**. Nein 🡪 Hatten Sie engen Kontakt zu einem Verdachtsfall?  1. Ja 🡪 Wann war der letzte Kontakt?  i. innerhalb der letzten 14 Tage  ii. länger als 14 Tage her  2. Nein | **a.** Yes 🡪 When was the most recent contact? Note: Please contact the hotline of your local health department. Health Department of Marburg: Phone +49 (0) 6421-40544  1. within the last 15 days  2. more than 15 days ago  **b.** No 🡪 Did you have close contact with a suspected Corona case?  1. Yes 🡪 When was the most recent contact?  i. within the last 15 days  ii. more than 15 days ago  2. No | a. Oui 🡪 À quand remonte le dernier contact? Remarque: veuillez contacter la hotline de votre service de santé local. Département de la Santé de Marburg: Tel.: +49 (0)6421-40544  1. au cours des derniers 15 jours  2. il y a plus de 15 jours  b. Non 🡪 Étiez-vous en contact étroit avec un cas suspect de Corona?  1. Oui 🡪 à quand remonte le dernier contact?  i. au cours des dernier 15 jours  ii. il y a plus de 15 jours  2. Non |
| 6. Haben Sie oder hatten Sie Fieber über 38,5°C? (in den letzten 5 Tagen) | 6. Do or did you have a fever above 38.5°C? (within the last 5 days) | 6. Avez-vous ou aviez-vous une fièvre supérieure à 38,5°C? (au cours des 5 derniers jours) |
| 1. Ja 2. Nein | 1. Yes 2. No | 1. Oui 2. Non |
| 7. Haben Sie Gliederschmerzen | 7. Do you have body aches? | 7. Avez-vous des courbatures? |
| 1. Ja 2. Nein | 1. Yes 2. No | 1. Oui 2. Non |
| 8. Haben Sie anhaltenden Husten? | 8. Do you have persistent cough? | 8. Avez-vous une toux persistante? |
| 1. Ja 🡪 Haben Sie trockenen Husten oder haben Sie Husten mit Auswurf (Schleim)? 2. Trockener Husten 3. Husten mit Auswurf 4. Nein | 1. Yes 🡪 Do you have dry cough or do you have cough with expectoration (mucous)?   1. Dry cough  2. Cough with expectoration   1. No | 1. Oui 🡪 Avez-vous une toux sèche ou avez-vous une toux avec flegme?   1. Toux sèche  2. Toux avec flegme   1. Non |
| 9. Haben Sie eine laufende oder verstopfte Nase? (Schnupfen) | 9. Do you have symptoms of common cold? | 9. Avez-vous des symptômes de rhume? |
| 1. Ja 2. Nein | 1. Yes 2. No | 1. Oui 2. Non |
| 10. Haben Sie Durchfall? | 10. Do you suffer from diarrhea? | 10. Avez-vous de la diarrhée? |
| 1. Ja 2. Nein | 1. Yes 2. No | 1. Oui 2. Non |
| 11. Haben Sie Übelkeit und Erbrechen? | 11. Do you suffer from nausea or vomiting? | 11. Avez-vous des nausées et des vomissements? |
| 1. Ja 2. Nein | 1. Yes 2. No | 1. Oui 2. Non |
| 12. Haben Sie Halsschmerzen? | 12. Do you have a sore throat? | 12. Avez-vous un mal de gorge? |
| 1. Ja 2. Nein | 1. Yes 2. No | 1. Oui 2. Non |
| 13. Haben Sie Kopfschmerzen? | 13. Do you have headaches? | 13. Avez-vous mal à la tête? |
| 1. Ja 2. Nein | 1. Yes 2. No | 1. Oui 2. Non |
| 14. Haben Sie Veränderungen der Geschmacks- oder Geruchswahrnehmung bemerkt? | 14. Did you notice changes in taste or smell? | 14. Avez-vous remarqué des changements de goût ou d'odeur? |
| 1. Ja 2. Nein | 1. Yes 2. No | 1. Oui 2. Non |
| 15. Haben Sie deutliche Luftnot in Ruhe? (Luftnot: Sprechen eines Satzes ist nur mit zusätzlichen Atemzügen möglich) | 15. Do you have shortness of breath at rest? (Shortness of breath: speaking a sentence is only possible with additional breaths) | 15. Avez-vous un essoufflement au repos? (Essoufflement: prononcer une phrase n'est possible qu'avec des respirations supplémentaires) |
| 1. Ja 🡪 Empfinden Sie die Luftnot als bedrohlich?   1. Ja  2. Nein   1. Nein 🡪 Kommen Sie außer Atem, wenn Sie mehr als 30 Meter gehen oder 10 Treppenstufen steigen?   1. Ja  2. Nein | 1. Yes 🡪 Do you experience the shortage of breath as threatening?   1. Yes  2. No   1. No 🡪 Do you get out of breath when you walk more than 30 meters or climb 10 stairs?   1. Yes  2. No | 1. Oui 🡪 Ressentez-vous le manque de soufflé menaçant?   1. Oui  2. Non   1. Non 🡪 Vous êtes essoufflé lorsque vous marchez sur plus de 30 mètres ou montez 10 marches?   1. Oui  2. Non |
| 16. Fühlen Sie sich schlapp oder abgeschlagen? | 16. Do you feel tired or worn out? | 16. Vous sentez-vous fatigué ou épuisé? |
| 1. Ja 2. Nein | 1. Yes 2. No | 1. Oui 2. Non |
| **Vorerkrankungen**  Risiko für die Möglichkeit eines schweren Covid-19 Verlaufs | **Previous diseases**  Risk of a severe Covid-19 course | **Maladies préexistantes**  Risque de possibilité d'un cours Covid-19 sévère |
| 17. Rauchen Sie oder haben Sie in den letzten 5 Jahren regelmäßig geraucht? | 17. Do you smoke or have you smoked regularly in the past 5 years? | 17. Fumez-vous ou avez-vous fumé régulièrement au cours des 5 dernières années? |
| 1. Ja 🡪 Wie viele Zigaretten rauchen Sie durchschnittlich pro Tag?   1. < 5  2. 5-10  3. 10-20  4. > 20   1. Nein | 1. Yes 🡪 How many cigarettes do you smoke per day on average?   1. < 5  2. 5-10  3. 10-20  4. > 20   1. No | 1. Oui 🡪 Combien de cigarettes fumez-vous en moyenne par jour?   1. < 5  2. 5-10  3. 10-20  4. > 20   1. Non |
| 18. Haben Sie eine Lungenerkrankung (COPD, Asthma bronchiale, Lungenfibrose, Silikose) | 18. Do you have a lung disease (COPD, bronchial asthma, pulmonary fibrosis, silicosis) | 18. Avez-vous une maladie pulmonaire (MPOC, asthme bronchique, fibrose pulmonaire, silicose) |
| 1. Ja 🡪 Welche Lungenerkrankung liegt bei Ihnen vor? (Mehrfachauswahl möglich)   1. COPD  2. Asthma  3. Lungenfibrose  4. Silikose  5. Sonstige, oben nicht benannt   1. Nein 2. Ich weiß es nicht | 1. Yes 🡪 Which pulmonary disease do you have? (multiple answers are possible)   1. COPD  2. Asthma  3. Pulmonary fibrosis  4. Silicosis  5. Others, not mentioned above   1. No 2. I do not know | 1. Oui 🡪 Quelle maladie pulmonaire avez-vous? (réponses multiples sont possibles)   1. MPOC  2. Asthme  3. Fibrose pulmonaire  4. Silicose  5. Autres   1. Non 2. Je ne sais pas |
| 19. Haben Sie Diabetes (Zuckerkrankheit)? | 19. Do you have diabetes? | 19. Avez-vous le diabète? |
| 1. Ja 2. Nein 3. Ich weiß es nicht | 1. Yes 2. No 3. I do not know | 1. Oui 2. Non 3. Je ne sais pas |
| 20. Haben Sie eine Kreislauferkrankung? (Bluthochdruck, Herzschwäche, Stent, Herzklappenfehler) | 20. Do you have a circulatory disease? (High blood pressure, heart failure, stent, heart valve defects) | 20. Avez-vous une maladie circulatoire? (Hypertension artérielle, insuffisance cardiaque, stent, anomalies valvulaires cardiaques) |
| 1. Ja 2. Nein 3. Ich weiß es nicht | 1. Yes 2. No 3. I do not know | 1. Oui 2. Non 3. Je ne sais pas |
| 21. Hatten Sie einen Schlaganfall oder eine Hirnblutung? | 21. Have you had a stroke or cerebral hemorrhage? | 21. Avez-vous eu une attaque cérébrale ou des hémorrhagies cérébrales? |
| 1. Ja 2. Nein 3. Ich weiß es nicht | 1. Yes 2. No 3. I do not know | 1. Oui 2. Non 3. Je ne sais pas |
| 22. Besteht bei Ihnen eine Tumorerkrankung? | 22. Do you have a cancer disease? | 22. Avez-vous une maladie de cancer? |
| 1. Ja 🡪 Haben Sie eine Strahlentherapie und/oder Chemotherapie in den letzten 3 Monaten erhalten?   1. Ja  2. Nein   1. Nein 2. Ich weiß es nicht | 1. Yes 🡪 Did you receive radiotherapy and/or chemotherapy during the last 3 months?   1. Yes  2. No   1. No 2. I do not know | 1. Oui 🡪 Avez-vous reçu une radiothérapie et/ou une chimiothérapie au cours des 3 derniers mois?   1. Oui  2. Non   1. Non 2. Je ne sais pas |
| 23. Leiden Sie an einer chronischen entzündlichen Erkrankung (Rheuma, Morbus Crohn oder ähnliches)? | 23. Do you suffer from a chronic inflammatory disease (rheumatism, Crohn's disease or other)? | 23. Souffrez-vous d'une maladie inflammatoire chronique (rhumatismes, maladie de Crohn ou similaire)? |
| 1. Ja 🡪 Nehmen Sie dauerhaft Cortison oder andere Immunsuppressiva ein? (Methotrexat, Cyclosporin, Biologicals, Mofetil etc.)   1. Ja  2. Nein  3. Ich weiß es nicht   1. Nein 2. Ich weiß es nicht | 1. Yes 🡪 Do you regularly take cortisone or other immunosuppressants? (Methotrexate, cyclosporin, biologicals, mofetil etc.)   1. Yes  2. No  3. I do not know   1. No 2. I do not know | 1. Oui 🡪 Prenez-vous de la cortisone ou d'autres immunosuppresseurs de façon permanente? (Méthotrexate, cyclosporine, produits biologiques, mofétil, etc.)   1. Oui  2. Non  3. Je ne sais pas   1. Non 2. Je ne sais pas |
| 24. Ist Ihre Nierenfunktion eingeschränkt? | 24. Is your kidney function impaired? | 24. Votre fonction rénale est-elle altérée? |
| 1. Ja 2. Nein 3. Ich weiß es nicht | 1. Yes 2. No 3. I do not know | 1. Oui 2. Non 3. Je ne sais pas |
| 25. Ist bei Ihnen eine Allergie bekannt? (z.B. Pollen, Medikamente, Nahrungsmittel etc.) | 25. Do you have an allergy? (e.g. pollen, medication, food, etc.) | 25. Avez-vous une allergie? (p. ex. pollen, médicaments, nourriture, etc.) |
| 1. Ja 2. Nein 3. Ich weiß es nicht | 1. Yes 2. No 3. I do not know | 1. Oui 2. Non 3. Je ne sais pas |
| 26. Haben Sie sich im Zeitraum von Oktober 2019 bis heute gegen Grippe impfen lassen? | 26. Have you been vaccinated against flu between October 2019 and today? | 26. Avez-vous été vacciné contre la grippe entre octobre 2019 et aujourd'hui? |
| 1. Ja 2. Nein 3. Ich weiß es nicht | 1. Yes 2. No 3. I do not know | 1. Oui 2. Non 3. Je ne sais pas |
| 27. Sind Sie gegen Masern geimpft oder haben Sie eine Maserninfektion durchlebt? | 27. Have you been vaccinated against measles or have you had a measles infection? | 27. Avez-vous été vacciné contre la rougeole ou avez-vous eu une infection rougeoleuse? |
| 1. Ja 2. Nein 3. Ich weiß es nicht | 1. Yes 2. No 3. I do not know | 1. Oui 2. Non 3. Je ne sais pas |
| 28. Wie lautet Ihre Postleitzahl? | 28. What is your ZIP code? | 28. Quel est votre code postale? |
| FREITEXT | FREE TEXT | TEXTE LIBRE |
